# *Babesia* spp. in ticks and wildlife in different habitat types of Slovakia

**DOI:** 10.1186/s13071-016-1560-z

**Published:** 2016-05-20

**Authors:** Zuzana Hamšíková, Mária Kazimírová, Danka Haruštiaková, Lenka Mahríková, Mirko Slovák, Lenka Berthová, Elena Kocianová, Leonhard Schnittger

**Affiliations:** Institute of Zoology, Slovak Academy of Sciences, Dúbravská cesta 9, 845 06 Bratislava, Slovakia; Institute of Biostatistics and Analyses, Faculty of Medicine and Faculty of Science, Masaryk University, Kamenice 3, 625 00 Brno, Czech Republic; Institute of Virology, Biomedical Research Center, Slovak Academy of Sciences, Dúbravská cesta 9, 845 05 Bratislava, Slovakia; Instituto de Patobiología, CICVyA, INTA-Castelar, 1686 Hurlingham, Prov. de Buenos Aires, Argentina; CONICET (National Research Council of Argentina), C1033AAJ Buenos Aires, Argentina

**Keywords:** Piroplasmida, *Babesia* spp., *Ixodes ricinus*, *Haemaphysalis concinna*, Rodents, Birds, Slovakia

## Abstract

**Background:**

Babesiosis is an emerging and potentially zoonotic disease caused by tick-borne piroplasmids of the *Babesia* genus. New genetic variants of piroplasmids with unknown associations to vectors and hosts are recognized. Data on the occurrence of *Babesia* spp. in ticks and wildlife widen the knowledge on the geographical distribution and circulation of piroplasmids in natural foci. Questing and rodent-attached ticks, rodents, and birds were screened for the presence of *Babesia*-specific DNA using molecular methods. Spatial and temporal differences of *Babesia* spp. prevalence in ticks and rodents from two contrasting habitats of Slovakia with sympatric occurrence of *Ixodes ricinus* and *Haemaphysalis concinna* ticks and co-infections of *Candidatus* N. mikurensis and *Anaplasma phagocytophilum* were investigated.

**Results:**

*Babesia* spp. were detected in 1.5 % and 6.6 % of questing *I. ricinus* and *H. concinna*, respectively. Prevalence of *Babesia*-infected *I. ricinus* was higher in a natural than an urban/suburban habitat. Phylogenetic analysis showed that *Babesia* spp. from *I. ricinus* clustered with *Babesia microti*, *Babesia venatorum*, *Babesia canis, Babesia capreoli/Babesia divergens,* and *Babesia odocoilei. Babesia* spp. amplified from *H. concinna* segregated into two monophyletic clades, designated *Babesia* sp. 1 (Eurasia) and *Babesia* sp. 2 (Eurasia), each of which represents a yet undescribed novel species. The prevalence of infection in rodents (with *Apodemus flavicollis* and *Myodes glareolus* prevailing) with *B. microti* was 1.3 % in an urban/suburban and 4.2 % in a natural habitat. The majority of infected rodents (81.3 %) were positive for spleen and blood and the remaining for lungs and/or skin. Rodent-attached *I. ricinus* (accounting for 96.3 %) and *H. concinna* were infected with *B. microti, B. venatorum, B. capreoli/B. divergens*, *Babesia* sp. 1 (Eurasia), and *Babesia* sp. 2 (Eurasia). All *B. microti* and *B. venatorum* isolates were identical to known zoonotic strains from Europe. Less than 1.0 % of *Babesia*-positive ticks and rodents carried *Candidatus* N. mikurensis or *A. phagocytophilum*.

**Conclusion:**

Our findings suggest that *I. ricinus* and rodents play important roles in the epidemiology of zoonotic *Babesia* spp. in south-western Slovakia. Associations with vertebrate hosts and the pathogenicity of *Babesia* spp. infecting *H. concinna* ticks need to be further explored.

**Electronic supplementary material:**

The online version of this article (doi:10.1186/s13071-016-1560-z) contains supplementary material, which is available to authorized users.

## Background

*Babesia* spp. are tick-transmitted hemoprotozoans infecting a number of mammalian and some bird species, and together with *Theileria* spp. they are referred to as piroplasmids (order Piroplasmida) [1]. Species of *Babesia* vary in their virulence and can cause babesiosis in humans and animals [2]. The first case of human babesiosis in Europe was reported from Croatia in 1957 [3]. Since then, the number of cases in Europe has increased [4, 5]. Three cases of human babesiosis have been reported from Slovakia since 1991 [6]. Based on classical taxonomy, piroplasmids include three groups: (i) *Theileria*, i.e. *Theileria capreoli* (Clade V as defined in [1]); (ii) *Babesia* (*sensu stricto*), i.e. *Babesia canis, Babesia venatorum, Babesia odocoilei, Babesia divergens* and *B. capreoli* (Clade VI as defined in [1])*;* and (iii) *Babesia* (*sensu lato*), i.e. *Babesia microti* (Clade I as defined in [1]) [1, 7]. Molecular phylogenetic analyses confirmed that *B. microti* is a species complex, consisting of genetically diverse isolates that fall into a number of different clades [8]. Within these clades, the zoonotic “Jena” type [9] and the non-zoonotic “Munich” type [10] can be discriminated between rodent isolates from Europe. Common causative agents of human babesiosis in Europe are *Babesia divergens* and the *B. divergens*-like species, *B. venatorum,* and *B. microti-*like species [2].

Ixodid ticks are the primary vectors of *Babesia* spp. Zoonotic species of *Babesia* are transmitted mostly by species of the genus *Ixodes. Ixodes ricinus* is a common tick species in Slovakia [11] and in some areas it is known to co-occur with other species, such as *Ixodes trianguliceps* [12], *Dermacentor reticulatus* [13] and *Haemaphysalis concinna* [11, 13]. The immature stages of *I. ricinus, H. concinna* and *Dermacentor* spp. ticks feed on small and medium-sized mammals and, in addition, immature *I. ricinus* and *H. concinna* ticks are ectoparasites of birds [14, 15]. In contrast, adults of these tick species parasitize medium and large-sized mammals. Large domestic and wild-living ruminants (e.g. cattle and roe deer), but also ticks, due to transovarial transmission, can serve as reservoirs for *B. divergens* and *B. venatorum.* Small mammals are reservoirs for the transtadially-transmitted *B. microti* [16, 17]. Some bird species can potentially contribute to the spread of piroplasmids by carrying infected ticks, infect ticks via infectious blood, or act as hosts for transmission of pathogens between ticks through co-feeding [18].

Data on the presence of piroplasmids and their medical and veterinary importance in Slovakia are rare and limited to a few studies. Some studies focused on *Babesia* spp. present in *I. ricinus* [13, 19] and rodents [12], while others dealt with *B. canis* infections in *D. reticulatus* ticks or dogs [20, 21]. Although the presence of piroplasmids in *H. concinna* was confirmed in neighbouring countries [22, 23], to our knowledge the competence of *H. concinna* to transmit *Babesia* parasites has not been studied in Slovakia.

Recently, the geographic area where piroplasmids have been detected in ticks and cases of babesiosis have been recognized has expanded and new species of *Babesia* have been found [23–25]. Therefore, local investigations are essential to assess the emergence of new parasites and the potential risk of human and animal diseases.

The main objective of this study was: (i) to investigate the presence and determine the prevalence and diversity of *Babesia* spp. in selected wild-living vertebrate hosts, focusing on rodents and birds, and on questing ticks and ticks feeding on rodents in two different habitat types of south-western Slovakia with sympatric occurrence of *I. ricinus* and *H. concinna* ticks; (ii) to assess ecological associations and phylogenetic relationships of the *Babesia* spp. found in ticks and vertebrate hosts in the study area; and (iii) to assess co-infections of *Babesia*-infected ticks and rodents with other microorganisms.

## Methods

### Study area, collection of ticks, trapping of rodents and birds, ethical approval

The two study sites are located in the Small Carpathian Mountains (south-western Slovakia) and differ in their habitat type. The first site (48.17–48.20 N, 17.07–17.10E) is characterized by significant human intervention and represents an urban/suburban habitat in Bratislava used for relaxing, cycling, dog walking, and jogging among others. The second site (48.37–48.38 N, 17.30–17.32E) is a natural habitat at Fúgelka represented by a non-fragmented forest, predominantly perambulated by hikers, foresters, and gamekeepers [for details, see 11]. Collection of questing ticks and trapping of rodents were performed as described previously [26]. In brief, questing ticks were collected from year 2011 to 2013 by dragging the vegetation and subsequently their species identity and life stage was determined. Rodents were livetrapped from year 2012 to 2014 by using Swedish bridge metal traps and sacrificed according to current laws of the Slovak Republic, approved by the Ministry of Environment of the Slovak Republic, Regional Environmental Office in Bratislava (licence ZPO-594/2012-SAB). Blood samples were obtained from *sinus orbitalis,* spleens, lungs, skin biopsy samples taken from ears (further as “skin”), and rodent-attached ticks were gathered from each rodent (at least five specimens of each tick species and life stage, respectively) for further analysis. Ornithological mist nets were used to trap wild-living birds in the urban/suburban habitat during 2012–2013. Each captured bird was identified, ringed, inspected for ectoparasites (data not shown) and blood samples were taken from the *vena ulnaris cutanea* before release as described in [27]. Birds were handled under the permission of the Ministry of Environment of the Slovak Republic, No. 9368/2011-2.2.

### DNA extraction

Genomic DNA was isolated from individual ticks and rodent tissues by using the Macherey-Nagel NucleoSpin® Tissue kit (Düren, Germany) according to the manufacturer’s instructions. Quantity and quality of the isolated DNA was assessed with a spectrophotometer Nanodrop 2000c and stored at -20 °C until further studied.

### PCR amplification and sequence analysis

DNA amplification by PCR was carried out following the protocol described by [28]. *Babesia* genus-specific BJ1 (5'-GTC TTG TAA TTG GAA TGA TGG-3') and BN2 (5'-TAG TTT ATG GTT AGG ACT ACG-3') primers were used to amplify a 450 bp region of the 18S ribosomal RNA gene. PCR reactions were carried out in a volume of 25 μl containing 5 μl of DNA template and 20 μl of PCR mix: 0.125 μl of HotStarTaq Plus DNA Polymerase (5 U/μl; Qiagen, Hilden, Germany), 0.5 μl of each primer (10 μM), 0.5 μl of dNTP (10 mM), 2.5 μl of Coral Load PCR buffer (containing 15 mM MgCl_2_), 1 μl of MgCl_2_ (25 mM) and 14.875 μl of nuclease free water. Negative as well as positive controls were included in each run. Amplification was performed in a BioRad t 100 thermal cycler (USA). The thermal cycle reaction consisted of an initial denaturation step (5 min at 95 °C), followed by 35 cycles of denaturation (1 min at 94 °C), annealing (1 min at 55 °C), and elongation (2 min at 72 °C). A final extended amplification step of 5 min at 72 °C was carried out. PCR products were separated by electrophoresis in a 1.5 % agarose gel and treated with GoodView™ Nucleic Acid stain (SBS Genetech, China) to be visualized by UV transillumination.

PCR positive samples were purified and analysed by sequencing with forward and reverse primers used for PCR amplification by Macrogen (Amsterdam, the Netherlands). Sequences were deposited in the GenBank database under accession numbers KU362887 − KU362905 and KU550676 − KU550699.

### Phylogenetic analysis

Determined 18S rRNA gene nucleotide sequences were used as query in a BLASTn search in order to identify and download most closely related 18S rRNA gene sequences of well-defined piroplasmid species from GenBank. In addition, 18S rRNA gene sequences of representative piroplasmid species were downloaded in order to allow species delineation in the phylogenetic analysis. A multiple alignment of the hypervariable region of 95 18S rRNA gene sequences comprising selected and analysed sequences including the 18S rRNA gene of *Cardiosporidium cionae* was done using MUSCLE [29]. Positions containing gaps and missing data were eliminated from the 514 nucleotide-alignment to finally result in 385 positions in the final dataset. After estimation of shape parameter, the K2 + G + I parameter model was applied to generate a maximum likelihood tree [30]. Phylogenetic analysis was carried out using the MEGA6 software [31].

### Statistical analyses

Differences in the prevalence of infection with *Babesia* spp. in questing ticks, ticks attached to rodents, and in rodents were analysed between habitats, years, and rodent species and genders applying Fisher’s exact test, supplemented with Mantel-Haenszel common odds ratio estimate and its 95 % confidence interval in cases when two prevalences were compared. Rodents positive for spleen, blood and/or lungs were considered *Babesia*-positive. The 95 % confidence intervals of the prevalences in questing ticks, rodent-attached ticks and rodents were computed using a bootstrap technique. Logistic regression was used to estimate the effect of habitat type and year on the probability of tick infection and the effect of habitat type, rodent species and gender on the probability of rodent infection. Backward stepwise method was used to find the set of variables significantly affecting the probability of tick and rodent infection. Tests for the significance of the effects in the model were performed via the Wald statistic. Results on the presence of *A. phagocytophilum* and *Candidatus* N. mikurensis (CNM) in the same questing ticks, and rodents available from previous studies [26, 32], were used to calculate the probability of co-infections with *Babesia* spp. and analyse the dependence of the microorganisms on the habitat type using Fisher’s exact test. Differences were considered significant at *P* < 0.05 in all tests. Statistical analyses were performed with IBM SPSS Statistics, version 22 [33] and Statistica software, version 12 [34].

## Results

### *Babesia* spp. in questing ticks

A total of 5057 *I. ricinus* (3158 nymphs and 1899 adults) and 91 *H. concinna* (59 nymphs and 32 adults) were examined, resulting in an overall *Babesia* spp. infection prevalence of 1.5 % (Table 1) and 6.6 % (Additional file 1: Table S1), respectively. The overall prevalence of *Babesia-*infected *I. ricinus* ticks was significantly higher in Fúgelka than in Bratislava (2.0 % *vs* 1.2 %; *P* = 0.022; OR = 1.7; CI: 1.1–2.7) (Table 1). Differences in prevalence of infection between sites were also significant for tick females (*P* = 0.016; OR = 10.4; CI: 1.2–89.5), but not for males (*P* = 0.560; OR = 0.6; CI: 0.2–2.1) and nymphs (*P* = 0.088; OR = 1.6; CI: 0.9–2.7) (Table 1). No significant differences were found between infection prevalence in *I. ricinus* nymphs and adults (Bratislava: *P* = 0.218; OR = 1.7; CI: 0.8–3.4; Fúgelka: *P* = 0.134; OR = 1.8; CI: 0.8–3.9). By comparing the prevalence of infection with *Babesia* spp. in *I. ricinus* between the three years (2011–2013), significant difference was revealed only for nymphs and for total prevalence in Bratislava (Table 1).

**Table 1:** Prevalence of *Babesia* spp. in questing *Ixodes ricinus* per site in 2011–2013

		2011		2012		2013		Fisher’s	Total	
Site		% (pos/ex)	95 % CI	% (pos/ex)	95 % CI	% (pos/ex)	95 % CI	exact test *P*	% (pos/ex)	95 % CI
Bratislava	Nymphs	0.9 (8/883)	0.3–1.6	4.1 (8/195)	1.5–7.2	1.3 (6/455)	0.4–2.6	0.007	1.4 (22/1533)	0.8–2.0
	Females	0.3 (1/367)	0–0.8	0 (0/61)	–	0 (0/156)	–	1.000	0.2 (1/584)	0–0.5
	Males	0.9 (4/437)	0.2–1.8	1.5 (1/68)	0–4.4	2.8 (5/177)	0.6–5.6	0.189	1.5 (10/682)	0.7–2.5
	Adults total	0.6 (5/804)	0.1–1.2	0.8 (1/129)	0–2.3	1.5 (5/333)	0.3–3.0	0.316	0.9 (11/1266)	0.4–1.3
	Total	0.8 (13/1687)	0.4–1.2	2.8 (9/324)	0.9–4.6	1.4 (11/788)	0.6–2.3	0.010	1.2 (33/2799)	0.8–1.6
Fúgelka	Nymphs	2.2 (23/1067)	1.3–3.1	3.4 (10/295)	1.4–5.4	1.5 (4/263)	0.4–3.0	0.319	2.3 (37/1625)	1.6–3.0
	Females	3.3 (5/150)	0.7–6.7	0 (0/59)	–	0 (0/76)	–	0.128	1.8 (5/285)	0.4–3.5
	Males	0.6 (1/164)	0–2.4	0 (0/82)	–	2.0 (2/102)	0–4.9	0.450	0.9 (3/348)	0–2.0
	Adults total	1.9 (6/314)	0.6–3.5	0 (0/141)	–	1.1 (2/178)	0–2.8	0.294	1.3 (8/633)	0.5–2.2
	Total	2.1 (29/1381)	1.4–2.9	2.3 (10/436)	0.9–3.7	1.4 (6/441)	0.5–2.5	0.554	2.0 (45/2258)	1.4–2.6
Total		1.4 (42/3068)	0.9–1.8	2.5 (19/760)	1.4–3.7	1.4 (17/1229)	0.7–2.0	0.086	1.5 (78/5057)	1.2–1.9

(pos/ex), number of positive/number of examined; 95 % CI, confidence interval

Overall prevalence of *Babesia* spp.-infected *H. concinna* ticks was higher in Bratislava compared to Fúgelka, but the difference was not significant (8.9 % *vs* 2.9 %; *P* = 0.400; OR = 3.3; CI: 0.4–29.8) (for details see Additional file 1: Table S1).

By comparing the two tick species, overall prevalence of infection with *Babesia* spp. was found to be significantly higher in *H. concinna* than in *I. ricinus* from Bratislava (8.9 % *vs* 1.2 %; *P* = 0.001; OR = 8.2; CI: 3.1–21.9). In contrast, no significant difference between the prevalence of infected *H. concinna* and *I. ricinus* was found at Fúgelka (2.9 % *vs* 2.0 %; *P* = 0.511; OR = 1.4; CI: 0.2–10.8).

**Fig. 1:**
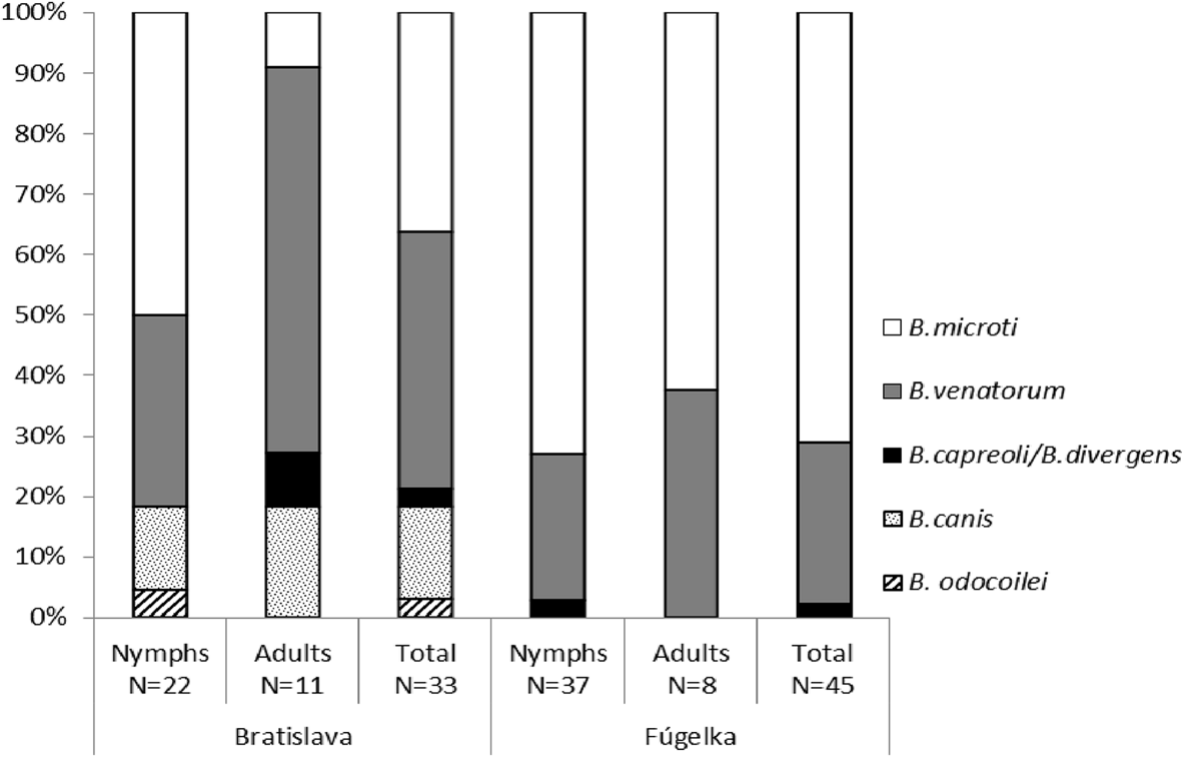
*Babesia* spp. in *Babesia*-infected questing *Ixodes ricinus* ticks in two different habitat types in south-western Slovakia. Bratislava, urban/suburban habitat; Fúgelka, natural habitat; N, number of examined *Babesia*-infected ticks

The occurrence of various species of *Babesia* in questing *I. ricinus* differed between habitats as well as between nymphs and adults (Fig. 1). The dependence of the occurrence of *Babesia* spp. on the habitat was significant (*P* = 0.002). Ticks infected with *B. microti* prevailed in the natural habitat (Bratislava: 27.3 %; Fúgelka: 72.7 %), whereas the proportion of ticks infected with *B. venatorum* was similar between both habitats (Bratislava: 53.8 %; Fúgelka: 46.2 %) (see Additional file 1: Table S2). *Babesia canis* (from four nymphs and one male) and *Babesia odocoilei* (from one nymph) were exclusively found in *I. ricinus* from Bratislava. *Babesia capreoli/B. divergens* was found in adult *I. ricinus* ticks from Bratislava and in one *I. ricinus* nymph from Fúgelka (Fig. 1). Furthermore, *Babesia* sp. 1 (Eurasia) (from four nymphs and one female from Bratislava) and *Babesia* sp. 2 (Eurasia) (from one male from Fúgelka) were found to infect questing *H. concinna* ticks.

The occurrence of *Babesia* spp. also differed significantly between years (*P* = 0.004). The proportion of *B. microti*-infected ticks was the higest in 2011 (50.0 %) and the lowest in 2013 (11.4 %). The proportion of *B. venatorum*-infected ticks was also the higest in 2011 (57.7 %) but it was the lowest in 2012 (7.7 %) (see Additional file 1: Table S3). There was also a significant difference in the proportion of tick developmental stages infected with *B. microti* and *B. venatorum* (*P* = 0.012). This was most obvious for *B. microti*, which was more prevalent in nymphs than in adults (86.4 % *vs* 13.6 %). In the other *Babesia* spp. the trend was not so strong (see Additional file 1: Table S4).

In addition to *Babesia* spp.*, Theileria* sp. DNA was detected in two *H. concinna* nymphs from Bratislava.

The analysis of simultaneous effects of habitat and year on the probability of the overall infection of *I. ricinus* with *Babesia* spp. by logistic regression resulted in a significant effect of habitat (Bratislava: parameter estimate B = -0.533, exp(B) = 0.587, *P* = 0.021). The variable removed by backward method was year. Considering only infections with *B. microti*, logistic regression confirmed the significant effect of habitat, with about three times lower probability of infection of ticks in the urban habitat (Bratislava: parameter estimate B = -1.064, exp(B) = 0.345, *P* = 0.002). In contrast, none of the variables were found to predict the infection of *I. ricinus* with *B. venatorum.*

### *Babesia microti* in rodents

Altogether, 606 rodents of six species (356 *Apodemus flavicollis*, 227 *Myodes glareolus*, 19 *Microtus arvalis,* 2 *Apodemus sylvaticus*, 1 *Microtus subterraneus,* 1 *Micromys minutus*) were screened for the presence of piroplasmids. *Babesia microti* was detected in spleen and/or blood and/or lungs of 1.3 % and 4.2 % of the examined rodents from Bratislava and Fúgelka, respectively (Table 2), with statistically significant difference between the two sites (*P =* 0.046; OR = 3.3; CI: 1.1–10.2). DNA of the parasite was also detected in lungs and skin biopsies from ears of rodents with positive spleens: in 3 lungs and 2 skin samples from rodents in Bratislava, and in 8 lungs and 7 skin samples from rodents in Fúgelka. Out of the 17 positive rodents, 47.1 % belonged to *A. flavicollis*, 47.1 % to *M. arvalis* and 5.8 % to *M. glareolus.* By comparing total *B. microti* infection prevalence between mice (the group comprises *A. flavicollis, A. sylvaticus* and *M. minutus*), *M. glareolus,* and *Microtus* spp. (the group comprises *M. arvalis* and *M. subterraneus*), a significant difference was determined (*P* < 0.001), with higher prevalence in *Microtus* spp. Overall, the parasite was found in 2.2 % (CI: 0.8–3.9 %) of mice, in 0.4 % (CI: 0.0–1.3 %) of *M. glareolus* and in 40.0 % (CI: 20.0–60.0 %) of *Microtus* spp. In addition, out of the rodents from Fúgelka (not included in the statistical analyses) that had negative spleen, blood and lungs, two *M. glareolus* females had positive skin.

**Table 2:** Prevalence of *Babesia microti* in rodents per species, gender and site

		Males		Females		Fisher’s	Total	
Site	Species	% (pos/ex)	95 % CI	% (pos/ex)	95 % CI	exact test *P*	% (pos/ex)	95 % CI
Bratislava	Mice^a^	4.1 (4/97)	1.0–8.2	0 (0/84)	–	0.125	2.2 (4/181)	0.6–5.0
	*M. glareolus*	0 (0/65)	–	0 (0/54)	–	–	0 (0/119)	–
	Total	2.5 (4/162)	0.6–4.9	0 (0/138)	–	0.127	1.3 (4/300)	0.3–2.7
Fúgelka	Mice^a^	3.1 (3/98)	0–7.1	1.3 (1/80)	0–3.8	0.629	2.2 (4/178)	0.6–4.5
	*M. glareolus*	1.9 (1/53)	0–5.7	0 (0/55)	–	0.491	0.9 (1/108)	0–3.7
	*Microtus* spp.^b^	75.0 (6/8)	37.5–100.0	16.7 (2/12)	0–41.7	0.019	40.0 (8/20)	20.0–60.0
	Total	6.3 (10/159)	2.5–10.1	2.0 (3/147)	0–4.8	0.089	4.2 (13/306)	2.0–6.5
Total		4.4 (14/321)	2.2–6.5	1.1 (3/285)	0–2.5	0.014	2.8 (17/606)	1.7–4.3

(pos/ex), number of positive/number of examined; 95 % CI, confidence interval; ^a^Mice comprise *Apodemus flavicollis*, *Apodemus sylvaticus* (1 female from Bratislava, 1 male from Fúgelka) and one *Micromys minutus* male from Fúgelka; ^b^ *Microtus* spp. comprises of *Microtus arvalis* and one *Microtus subterraneus* female

The prevalence of infection with *B. microti* was significantly higher in male rodents (4.4 % *vs* 1.1 %; *P* = 0.014; OR = 4.3; CI: 1.2–15.1), but no significant differences were found between genders of individual species except for *Microtus* spp. in Fúgelka (Table 2).

Significant difference in the overall prevalence of infection with *B. microti* was determined between years: 3.7 and 25.0 % of rodents were found to be positive in 2012 and 2013, respectively, but no rodent was found to be infected in 2014 (*P* = 0.004) (Table 3). Considering habitat, the difference between years was statistically significant in Fúgelka, but not in Bratislava (Table 3).

**Table 3:** Prevalence of *Babesia microti* in rodents per site in 2012–2014

	2012		2013		2014		Fisher’s exact test *P* ^a^
Site	% (pos/ex)	95 % CI	% (pos/ex)	95 % CI	% (pos/ex)	95 % CI	
Bratislava	1.1 (2/185)	0–2.7	33.3 (2/6)	0–66.7	0 (0/109)	–	0.532
Fúgelka	5.9 (13/222)	2.7–9.5	0 (0/2)	–	0 (0/82)	–	0.023
Total	3.7 (15/407)	1.7–5.7	25.0 (2/8)	0–62.5	0 (0/191)	–	0.004

(pos/ex), number of positive/number of examined; 95 % CI, confidence interval; ^a^only years 2012 and 2014 were compared

Simultaneous effects of habitat, rodent species, and gender on the probability of infection with *B. microti*, analysed by logistic regression, resulted in a significant effect of species (mice: parameter estimate B = -4.142, exp(B) = 0.016, *P* < 0.001; *Myodes*: parameter estimate B = -5.755, exp(B) = 0.003, *P* < 0.001) and gender (males: parameter estimate B = 2.345, exp(B) = 10.431, *P* = 0.004). The variable removed by backward method was habitat (see Additional file 1: Table S5). The probability of infection was the highest for *Microtus* spp., and the risk of infection of rodent males was ten times higher than that of females.

### *Babesia* spp. in rodent-attached ticks

In total, 2003 engorged ixodid ticks were collected from rodents: 1089 and 840 *I. ricinus,* 30 and 39 *H. concinna* from Bratislava and Fúgelka, respectively, 4 *I. trianguliceps* from Bratislava and 1 *D. reticulatus* from Fúgelka. Altogether, 1140 (695 and 445 from Bratislava and Fúgelka, respectively) rodent-attached ticks were screened: 1075 *I. ricinus* (1044 larvae, 28 nymphs, 3 females), 60 *H. concinna* (56 larvae, 4 females), 4 *I. trianguliceps* (2 larvae, 2 nymphs), and 1 *D. reticulatus* larva. Piroplasmids were detected in *I. ricinus* (immature stages and 1 female) and *H. concinna* (larvae and females), but not in *I. trianguliceps* and *D. reticulatus* (Table 4)*.*

**Table 4:**
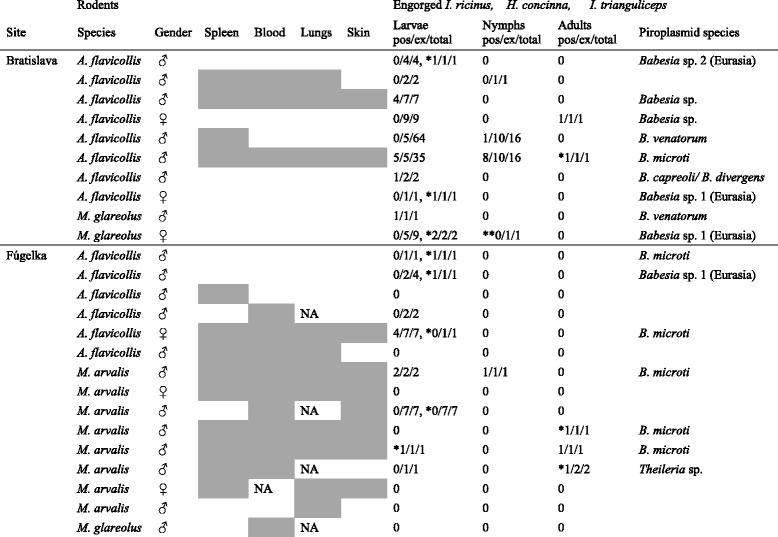
Dissemination of *Babesia microti* in infected rodents and infestation of rodents with *Babesia* (*Theileria*)-positive ticks

Grey, *Babesia*-positive; white, *Babesia*-negative; pos/ex/total, number of positive (positive by amplification of the 18S rRNA gene fragment/number of examined ticks/number of total ticks infesting a rodent); NA, not available. The table displays all *B. microti-*positive rodents (infested and uninfested with ticks) and out of the *Babesia*-negative those specimens which were infested with *Babesia* (*Theileria*)-positive ticks. * *H. concinna*, ** I. trianguliceps

Thirty-eight out of 1140 (3.3 %; CI: 2.3–4.5 %) rodent-attached ticks were positive for *Babesia* spp., whereby 30 of them were collected from *B. microti*-positive rodents and the remaining from *Babesia*-negative specimens (Table 4). Individual *B. microti*-positive rodents carried 1 to 14 *Babesia-*positive ticks, whereby 9 of 300 (3.0 %; CI: 1.3–5.3 %) and 6 of 306 (2.0 %; CI: 0.7–3.6 %) rodents carried *Babesia*-positive ticks in Bratislava and Fúgelka, respectively. The prevalence of infection in rodent-attached ticks did not differ significantly between the two sites (*P* = 0.445; OR = 1.5; CI: 0.5–4.4). In addition to the most prevalent *B. microti,* a few rodent-attached *I. ricinus* ticks carried *B. venatorum* and *B. capreoli/B. divergens.* In rodent-attached *H. concinna, B. microti, Babesia* sp. 1 (Eurasia), *Babesia* sp. 2 (Eurasia) and *Theileria* sp. were detected (Table 4).

### Co-infections in ticks and rodents

With regard to co-infections and prevalence patterns, we analysed results for *Babesia* spp. and data from previous studies on prevalences of *A. phagocytophilum* and CNM in ticks and rodents [26, 32]. Out of the 3874 questing *I. ricinus* screened for the presence of the three microorganisms, co-infection of *Babesia* spp. and *A. phagocytophilum* was detected in two ticks (0.05 %; one male infected with *B. venatorum* and one nymph infected with *B. canis* from Bratislava). Co-infection of *Babesia* spp. and CNM was found in three ticks (0.08 %; nymphs from Fúgelka, two infected with *B. microti* and one infected with *B. venatorum*). Triple infections were not detected. Comparison of the proportions of ticks infected with the microorganisms revealed significant differences between the two habitats (*P* < 0.001). Ticks infected with *Babesia* spp. (62.3 %) and CNM (65.5 %) prevailed in the natural habitat, and ticks infected with *A. phagocytophilum* (74.7 %) prevailed in the urban/suburban habitat (see Additional file 1: Table S6).

Altogether, five out of the 606 examined rodents (0.83 %; one *A. flavicollis* male from Bratislava, one *A. flavicollis* female, two *M. arvalis* females and one *M. arvalis* male from Fúgelka) were co-infected with *B. microti* and CNM. The proportions of rodents infected with the two microorganisms did not differ significantly between the two habitats (*P* = 1.000). Rodents infected with *B. microti* (75.0 %; 9 out of 12) and CNM (75.0 %; 27 out of 36) prevailed in the natural habitat. No co-infection of *B. microti* and *A. phagocytophilum* was observed.

Considering rodent-attached ticks, only co-infection of *B. microti* and *A. phagocytophilum* was detected in three *I. ricinus* nymphs feeding on a *B. microti-*positive *A. flavicollis* male from Bratislava. The remaining infected engorged ticks carried only one microorganism.

### *Babesia* spp. in birds

In total, 58 blood samples from birds representing 11 species were screened for *Babesia* spp. (Appendix 1). None of the birds was found to be infected.

### Phylogenetic analysis

**Fig. 2:**
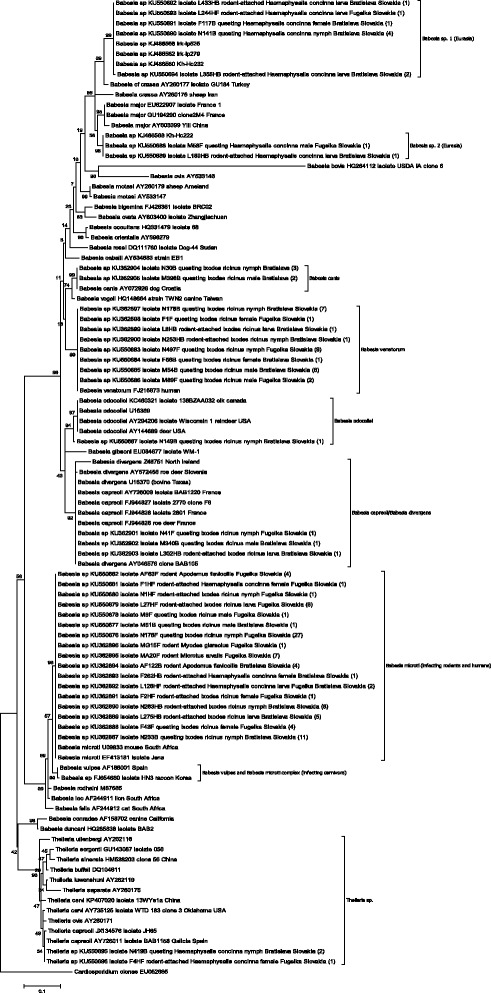
Phylogenetic tree of hypervariable 18S rRNA gene sequences of *Babesia* and *Theileria* parasites using maximum likelihood. The sequence of each isolate is labelled with its gene accession number, isolate designation, host (questing tick, rodent-attached tick, and rodent), and geographic origin. The bootstrap values based on 1,000 replicates are displayed next to the branches. The tree is rooted using *Cardiosporidium cionae* as outgroup [1]. Wherever applicable, the number of identical sequences of a given isolate type is given. All clades marked by brackets display a highly significant bootstrap value (≥ 85). The evolutionary distance is shown in the units of the number of base substitutions per site

All piroplasmid-positive PCR products from questing ticks, ticks attached to rodents, and rodents originating from both study sites were sequenced and are listed in Additional file 1: Table S7. The phylogenetic analysis shows that the majority of isolates segregate with a highly significant bootstrap into clades of *Theileria* sp., *B. microti*, *B. venatorum*, *B. canis, B. odocoilei,* and a *B. capreoli/B. divergens* clade, respectively. However, some isolates segregate into two novel clades strongly suggesting that they represent previously unrecognized species designated in this study as *Babesia* sp. 1 (Eurasia) and *Babesia* sp. 2 (Eurasia), respectively (Fig. 2). In the phylogenetic analysis, all identified *B. microti* isolate sequences clustered with strong support into a single clade with the Jena/Germany genotype (Fig. 2). Sequences of 17 isolates (KU362887−KU362896 and KU550676−KU550682; from questing and rodent-attached *I. ricinus,* rodent-attached *H. concinna* and from rodents) corresponding to 114 analysed 18S rRNA gene sequences show a 100 % sequence identity with the pathogenic *B. microti* Jena/Germany genotype (EF413181). Other isolates displaying a 100 % sequence identity not included in the tree analysis are a *B. microti* isolate from a rodent (KJ649297) and from a questing *I. ricinus* from Slovakia (KJ649287).

18S rRNA gene sequences of eight isolates (KU362897−KU362900 and KU550683−KU550686; from questing and rodent-attached *I. ricinus*) corresponding to 28 analysed nucleotid sequences segregated with a significant bootstrap into a single clade with the *B. venatorum* genotype (FJ215873) known to cause zoonotic babesiosis in Europe. Other deposited sequences found to be 100 % identical to those of this study were a *B. venatorum* isolate from *I. ricinus* from Slovakia (KJ152840) and the Czech Republic (KJ465867), but also to a *B. venatorum* isolate identified in *Ixodes persulcatus* from Mongolia (KR493908).

18S rRNA gene sequences from isolates from five questing *I. ricinus* (KU362904 and KU362905) segregated with highly significant support into a clade with *B. canis* isolate AY072926 identified in a dog in Croatia. Isolate sequences identified in the GenBank database that were found to be 100 % identical represent *B. canis* from a naturally infected domestic dog from Poland (KT844907) and an isolate identified from *D. reticulatus* from Russia (AY649326).

18S rRNA gene sequences isolated from questing *I. ricinus* (KU362901 and KU362902) and a sequence from a rodent-attached *I. ricinus* (KU362903) clustered in a strongly supported clade with sequences of *B. capreoli* and *B. divergens*. Identified sequences were also found to be 100 % identical with sequences deposited in the GenBank database from *B. capreoli* from *I. ricinus* from the Czech Republic (KJ465869) and with a strain isolated from roe deer from Germany (JX627353), and 99 % identical with *B. divergens* isolated from a roe deer from Slovenia (AY572456).

Four isolates from questing and rodent-attached *H. concinna* were placed into a strongly supported single clade with isolate *Babesia* sp. Kh-Hc232 from *H. concinna* (KJ486560) and *Babesia* sp. Irk-Ip525 from *I. persulcatus* (KJ486566)*.* This clade represents a novel species of *Babesia* here designated as *Babesia* sp. 1 (Eurasia). The 18S rRNA gene sequence of the isolate KU550694 differs from isolate sequences KU550690−KU550693 by a single base pair.

Sequences isolated from questing and rodent-attached *H. concinna* ticks (KU550688 and KU550689) appeared in a strongly supported clade with the sequence of *Babesia* sp. Kh-Hc222 strain from *H. concinna* identified in Russia (KJ486568). The sequences in this clade have been designated as *Babesia* sp. 2 (Eurasia) as they most probably represent an additional novel species.

Other 18S rRNA gene sequences isolated in this work from *I. ricinus* (KU550687) clustered in a strongly supported clade with *B. odocoilei* isolates U16369, KC460321, AY294206, and AY144689 that are 99 % identical with the former. However, the sequence KU550687 is also placed with strong support as sister to this clade and may represent a geographical variant of this species. Other sequences identified in GenBank that are 99 % identical with KU550687 are, e.g. the Norwegian strain *Babesia* sp. OO-2012 (JX083978) from *I. ricinus*, the Austrian strain *Babesia* cf. *odocoilei* (JN543180) from red deer, and the German strain *B. odocoilei* (JX679176) from *I. canisuga*.

18S rRNA gene sequences isolated from questing *H. concinna* (KU550695 and KU550696) belong to the genus *Theileria* as evidenced by their placement into this strongly supported clade. Placement of species of *Theileria* within this clade displays non-significant bootstraps and thus the species identity of KU550695 and KU550696 cannot be finally verified. However, these are most closely related to *Theileria capreoli* isolates JX134576 and AY26011 displaying a 99 % identity. Isolate sequence KU550695 is 100 % identical to GenBank deposited sequences of a *Theileria* sp. isolate from fox from Croatia (HM212629) and to a strain from roe deer from Spain (DQ866842) while sequence (KU550696) is 100 % identical with a *Theileria* sp. isolate from red deer from Poland (DQ520836). The 18S rRNA gene sequence of isolate KU550695 differs from isolate sequence KU550696 by a single base pair.

### Other Apicomplexa detected in ticks and rodents

Isolates from four questing *I. ricinus* showed identity with *Hepatozoon canis* DNA. *Hepatozoon* spp. DNA was also identified in 26 *M. glareolus* and one *A. flavicollis* (manuscript in preparation).

18S rRNA gene sequences from six isolates from rodent skin biopsies showed identity with corresponding sequences of *Sarcocystis* spp. Four identical sequences from *M. arvalis* (KU550697) revealed a 96 % identity to the 18S rRNA gene sequence of *Sarcocystis* sp. from the large oriental voles (*Eothenomys miletus*) from China (KF309698 and KF309699). One sequence from *M. glareolus* (KU550699) showed a 97 % identity to the same strains (KF309698 and KF309699). One sequence obtained from *M. glareolus* (KU550698) showed a 97 % identity to the sequence of *Sarcocystis rodentifelis* from a rodent in the Czech Republic (AY015111), *Sarcocystis rileyi* from a mallard duck (*Anas platyrhynchos*) from Lithuania (HM185742), *Sarcocystis speeri* from an opossum (*Didelphis virginiana*) from Argentina (KT207459), and *Sarcocystis* sp. from a brown bear (*Ursus arctos*) from USA (EF564590).

## Discussion

There are only a few studies of piroplasmid parasites associated with ticks and wildlife from Slovakia. This calls for further investigations on the distribution and diversity of piroplasmid species and their relevance to public and animal health in the region. In the present study, questing and rodent-attached ticks, rodents and birds were screened using molecular methods for the presence of *Babesia* spp. to investigate the vector – host – pathogen associations in the urban/suburban and natural habitats of south-western Slovakia. The study area is characteristic of a sympatric occurrence of *I. ricinus* and *H. concinna* ticks and a great diversity of wildlife [11].

We found 1.5 % of questing *I. ricinus* and 6.6 % of *H. concinna* ticks to be infected with *Babesia* parasites. In previous studies from Slovakia, the prevalence of infection with *Babesia* spp. in questing *I. ricinus* was similar and varied from 0.4 to 2.7 % [12, 13, 19] while no reports exist on the presence of piroplasmids in questing *H. concinna*. Generally, *Babesia* spp. prevalences from 0.4 to 2.7 % have been reported for questing *I. ricinus* in temperate latitudes of Europe [28, 35–43]. Yet at particular sites 4.6 to 9.6 % of questing *I. ricinus* were found to be infected with *Babesia* spp. [17, 44]. Relationships between the prevalence of infection with *Babesia* spp. of ticks and habitat type could be determined at several sites. Similar to our findings, a significantly higher proportion of *Babesia*-infected *I. ricinus* was found in a natural habitat than an urban area in Germany [40], but no *Babesia* spp. were found in *I. ricinus* from an urban habitat in the Czech Republic [42]. In contrast to *I. ricinus,* the overall prevalence of *Babesia*-infected *H. concinna* from our study was higher in the urban/suburban habitat than in the natural habitat. We assume that the observed variations in the overall prevalence of infection with *Babesia* spp. between sites and tick species are associated with the vector competence of ticks for particular species of *Babesia* and the presence and abundance of competent reservoir hosts.

*Babesia microti* and *B. venatorum* as emerging zoonotic species and in some studies also *B. divergens,* have frequently been detected in questing *I. ricinus* in Europe [12, 17, 28, 38–40, 42, 43]. In previous reports from Slovakia, *B. microti* was the most common species detected in field-collected *I. ricinus* ticks [12, 19]. Our results confirmed these observations. The parasite significantly prevailed in ticks from a natural habitat, as has been also reported in a study from Germany [40].

Phylogenetic analysis of partial 18S rRNA gene sequences from our study revealed their identity to those of the zoonotic *B. microti* Jena/Germany genotype. Zoonotic *B. microti* genotypes have been found to be associated with microtine rodents and shrews [45, 46]. In our study, *B. microti* was the only species detected in rodents and the same *B. microti* strain was also identified in questing and rodent-attached ticks. The overall prevalence of infection in rodents as reported by us is in accordance with previous findings from eastern Slovakia [12]. Prevalence of infection varying from 1.4 up to 27.2 % was reported for rodents from other European countries [17, 45, 47–51].

*Babesia microti*-like piroplasmids were previously detected in small mammals from central Europe based on morphological studies of blood and tissue preparations. For example, a 0.4 % prevalence of infection was determined in bank voles (*M. glareolus*) in former Czechoslovakia [52]. Relatively low proportions of bank voles were found infected by applying molecular methods in the present study (0.4 %) as well as in eastern Slovakia (1.1 %) [12], although detection rates of blood parasites would be expected to be higher when using molecular methods rather than microscopic examinations [50]. In other central European countries, prevalence of infection with *B. microti* varied in bank voles from 0.0 to 4.9 % [17, 50, 51, 53], whereas 6.1 to 15.9 % of bank voles were found to be infected in Slovenia and Croatia [45, 49]. In the study at hand, the single *B. microti*-infected bank vole was positive for blood, but not infested by ticks. Parasite DNA was also detected in the skin of two other bank voles, suggesting local infections.

Previous studies on the yellow-necked mice (*A. flavicollis*) in central Europe reported a prevalence of *B. microti* infection ranging from 0 to 1.6 % [17, 47, 51, 53] comparable to findings from eastern Slovakia [12] and also to our results. In contrast, higher percentages (11.8 to 16.2 %) of *B. microti*-infected mice were found in Slovenia and Croatia [45, 49].

Voles of the genus *Microtus* are considered to be the main reservoirs of *B. microti* in natural foci of Europe. Prevalences of infection from 8.3 to 14.3 % were reported for the common vole (*M. arvalis*) from different European sites [47, 51, 53]. A high proportion of infected common voles (40.0 %) was also confirmed in our study whereas none or only 0.7 % of the common voles were found to be infected in other sites of Slovakia [12, 52].

The presence of *B. venatorum,* a species recognized as a human pathogen [54], was confirmed in one third of *Babesia-*positive questing *I. ricinus* from our study. The occurrence of this parasite in questing ticks has been reported from a few sites of Slovakia only recently [13, 55, 56]. In contrast to *B. microti* infections, the proportion of ticks infected with *B. venatorum* did not differ between the two explored habitats. The roe deer, suggested to be the main reservoir host of this parasite species, [57] is present in both studied habitats. As transovarial transmission of *B. venatorum* has been demonstrated [58], it is probably also maintained in natural foci of Slovakia through its vector tick.

*Babesia capreoli/B. divergens* accounted for 2.6 % of the *Babesia*-positive *I. ricinus* ticks. The *B. divergens*-like hypervariable 18S rRNA gene region analyzed in our study showed identity with those of *B. capreoli* and *B. divergens* of which *B. divergens* has been demonstrated to be a zoonotic species [59]. Although cattle are considered the principal host of *B. divergens*, infections were also detected in deer assumed to function as reservoir host [60–62]. The presence of *B. divergens* in *I. ricinus* was noted once in Slovakia using the Reverse Line Blot Hybridization method [19]. In contrast, *B. capreoli*, a parasite of roe deer which differs marginally in the 18S rRNA gene sequence from *B. divergens*, is unable to infect humans [63]. To our knowledge, there are no published data on the presence of this species in Slovakia.

*Babesia odocoilei*, known to parasitize American white-tailed deer (*Odocoileus virginianus*) and to cause babesiosis in cervid and occasionally in bovid species in Europe [62, 64–66], was found in a questing *I. ricinus* nymph in our study. Related genotypes, e.g. *Babesia* cf. *odocoilei*, have been found to infect *I. ricinus* in Europe [39, 44], but their zoonotic potential is unknown.

The presence of *B. canis,* the most frequent causative agent of canine babesiosis in central Europe, has previously been shown in *D. reticulatus* ticks of Slovakia [20] and also in blood samples from naturally infected dogs [21]. Importantly, we report here for the first time *B. canis* infection of *I. ricinus* from Slovakia. Thus, we confirm the previous conjecture of the infection of *I. ricinus* with *B. canis* as noted in a study from Poland [67, 68]. However, our finding does not allow us to draw conclusions on the competence of *I. ricinus* as a vector of this parasite. We assume the *B. canis* DNA identified in *I. ricinus* ticks from the urban habitat may have originated from blood meals taken from infected dogs.

In the present study, *B. microti-*positive engorged *I. ricinus* ticks were sampled from infected rodents, but the parasite was also detected in a few larvae that fed on uninfected rodent specimens. Interestingly, we also found a few semi-engorged *B. microti*-positive *I. ricinus* females attached to rodents, however, it is uncertain if they can complete feeding on these hosts, as they generally prefer medium-sized to large mammalian hosts [69]. *Babesia microti*-positive *I. ricinus* larvae and nymphs were also found on rodents captured in eastern Slovakia [12] and Switzerland, where the pathogen was detected in xenodiagnostic ticks from infected bank voles [70]. In addition, we detected *B. microti* in a few *H. concinna* ticks feeding on infected rodents and in one larva collected from an uninfected yellow-necked mouse. There are no GenBank data on *B. microti* isolates from *H. concinna* and to the best of our knowledge, infection of this tick species by the parasite has not been reported. *Babesia microti* is thought to be incompetent for transovarial transmission in ticks [16, 71] and the positivity of tick larvae might have resulted while feeding on infected hosts or co-feeding with infected ticks. Accordingly, we assume that the positive *H. concinna* larva collected from an uninfected yellow-necked mouse acquired the infection via feeding on an infected host and, after interruption, subsequently attached to an uninfected rodent. Our results particularly underline the potential reservoir role of *A. flavicollis* and *M. arvalis* for *B. microti* at our study sites. In addition to *B. microti*, rodent-attached *I. ricinus* infected with *B. venatorum* and *B. capreoli/B. divergens* were found. As vertical transmission has been described for the latter two species of *Babesia* [7], we assume that these tick specimens acquired the infection from an infected female.

A number of *Babesia* spp. genetic variants that are closely related to small ruminant piroplasmids, *Babesia crassa, Babesia* cf. *crassa, Babesia motasi*, and to the cattle-infecting *Babesia major* have been found in *H. concinna* in Europe and Asia [1]. *Babesia motasi* is known as an agent of mild sheep and goat babesioses in various countries of Europe, Africa and Asia, while *B. crassa* and *B. crassa*-like piroplasmids have been detected in sheep blood in Iran and Turkey, and *B. major* in cattle from Europe and Asia [1]. In the inferred phylogenetic tree, *Babesia* spp. sequence variants identified in *H. concinna* from Slovakia clustered in two strongly supported monophyletic clades suggesting that each represents a novel species designated *Babesia* sp. 1 (Eurasia) and *Babesia* sp. 2 (Eurasia), respectively. Our results corroborate a wide distribution of these two novel piroplasmid species in *H. concinna* from Europe and Asia supporting recent findings [23, 72]. Their presence across a large area may be related to the broad geographic distribution of *H. concinna* in Eurasia and connection of habitats via longitudinal migration of birds which are known to be preferred hosts of this tick species [14, 15].

Piroplasmids of the genus *Theileria* found in *H. concinna* in the present study are known to infect a broad spectrum of free-ranging ungulates in neighbouring countries of Slovakia as well as in other regions of Europe [22, 23, 73]. The same or closely related strains of *Theileria* sp. were found in questing *H. concinna* from Austria [22], Hungary [23] and in rodent-attached and questing *H. concinna* from our study. The presence of *Theileria* sp. in *H. concinna* from Slovakia indicates that the tick species may be a suitable vector of species of *Theileria* in this region.

Detection of *Hepatozoon* spp. and *Sarcocystis* spp. DNA in rodents support former findings demonstrating that rodents are hosts of a large spectrum of apicomplexan parasites [17]. However, further molecular analyses of the isolates obtained during our study are necessary to reveal their identity with species of *Hepatozoon* and *Sarcocystis*.

Birds contribute to the geographic distribution of various tick-borne pathogens and serve as their hosts. Although there are no confirmed infections with *Babesia* spp. in birds from Europe, *B. microti, B. venatorum,* and *B. divergens* have been found in ticks infesting birds [71, 74–76]. The occurrence of *Babesia* spp. in ticks (especially in larvae) from birds suggested that birds may be able to infect ticks, at least in the case of *B. microti*, a species considered not to be transmitted transovarially. We did not find any *Babesia*-positive blood sample from birds and thus our results support previous findings that *Babesia* spp. associated with mammals do not infect birds [18]. Although studies on this topic are lacking, birds may act as carriers for *Babesia-*infected ticks contributing to the dispersal of the parasites in Europe [75, 76].

Co-infections with other microorganisms were detected in less than 0.1 % of the examined *Babesia*-infected *I. ricinus* ticks. This result agrees with findings reported from other European countries on co-infection rates depending on the habitat and varying from 0.0 to 1.8 % for *Babesia* spp. and *A. phagocytophilum* [17, 35, 68, 77] and from 0.02 to 1.8 % for *Babesia* spp. and CNM [78, 79]. The low co-infection rate (0.05 %) for *Babesia* spp. (with *B. microti* prevailing) and *A. phagocytophilum* in ticks reported in this study is not surprising as the two microorganisms seem not to share the same reservoir hosts in the investigated area [26]. In contrast, *B. microti* and CNM have been found to be associated with rodents [16, 32, 45, 46, 78, 80]. Accordingly, we would have expected to observe higher co-infection rates than determined in ticks (0.08 %) and rodents (0.83 %) in the context of this study. Generally, the microbiome of ticks was found to be very complex and rodents can carry a wide variety of microorganisms [81]. Information about the relationships (antagonism, mutualism) between particular microorganisms within the tick and the body of the vertebrate host as well as between microorganisms, ticks and the immune system of the reservoir host are scarce. We assume that the observed co-infection rates are the result of the complexity of the microorganisms – vector – host associations.

## Conclusions

This study employed molecular tools to detect the presence of *Babesia* spp. in ticks, rodents, and birds from two contrasting habitat types in the Small Carpathian Mountains in south-western Slovakia. Different *Babesia* spp. were found to be distributed in the tick populations of urban/suburban and natural habitats of Slovakia and rodents were found to play an important role as reservoirs of *B. microti. Babesia microti* and *B. venatorum* genotypes*,* identical with respective known zoonotic strains from Europe, dominated in questing and rodent-attached *I. ricinus*. In addition, *B. capreoli/B. divergens* and *B. odocoilei* were detected in questing *I. ricinus*. The occurrence of these *Babesia* spp. suggests that ungulates present in the study area may act as their reservoir hosts. *Ixodes ricinus* may occasionally acquire infection with *B. canis,* probably by feeding on infected dogs. Importantly, we corroborate previous reports of the existence of two novel piroplasmid species referred to as *Babesia* sp. 1 (Eurasia) and *Babesia* sp. 2 (Eurasia) which were both identified in questing and rodent-attached *H. concinna* ticks. Our results demonstrate a high diversity of *Babesia* spp. circulating in *I. ricinus* and *H. concinna* ticks. Important findings of this study are (i) the first detection of *B. canis* and *B. odocoilei* in questing *I. ricinus,* (ii) the detection of two novel species of *Babesia*, and (iii) the detection of a *Theileria* sp. in *H. concinna* in Slovakia. We demonstrate that *A. flavicollis* and *M. arvalis* may play a critical role in *B. microti* transmission supporting the hypothesis that the most competent reservoirs are voles of the genus *Microtus*, particularly *M. arvalis*. In contrast, *M. glareolus* seems of secondary importance for the circulation of *B. microti*. Although all studied birds were found to be *Babesia*-negative, they may represent potential hosts of infected *I. ricinus* and *H. concinna* ticks and thus contribute to the transmission and geographical spread of *Babesia* spp*.* Further research is needed to explore the vertebrate hosts of the yet undescribed novel species of *Babesia* and *Theileria* infecting *H. concinna,* and to determine their pathogenic potential for humans and animals.

## Appendix

**Table 5:** Bird species tested for *Babesia* spp.

Bird species	Count
European green woodpecker (*Picus viridis*)	2
Great spotted woodpecker (*Dendrocopos major*)	4
European robin (*Erithacus rubecula*)	2
Common blackbird (*Turdus merula*)	2
Eurasian blackcap (*Sylvia atricapilla*)	1
Spotted flycatcher (*Muscicapa striata*)	1
Great tit (*Parus major*)	25
Eurasian blue tit (*Cyanistes caeruleus*)	6
Willow tit (*Parus montanus*)	5
Eurasian nuthatch (*Sitta europaea*)	9
Common chaffinch (*Fringilla coelebs*)	1

## Additional file

Additional file 1: Table S1. Prevalence of *Babesia* spp. in questing *Haemaphysalis concinna* per site in 2011–2013. **Table S2.** Occurence of *Babesia* spp. in questing *Ixodes ricinus* in Bratislava and Fúgelka. **Table S3.** Occurence of *Babesia* spp. in questing *Ixodes ricinus* in 2011–2013. **Table S4.** Occurence of *Babesia* spp. in questing *Ixodes ricinus* males, females and nymphs. **Table S5.** Variables remaining in the best selected model for *Babesia microti* prevalence in rodents. **Table S6.** Occurence of *Babesia*, *Candidatus* N. mikurensis and *Anaplasma phagocytophilum* in questing *Ixodes ricinus.* **Table S7.** Accession numbers of Apicomplexa 18S rRNA gene sequences (PDF 238 kb)
