# Supplementary material for: Babesia spp. in ticks and wildlife in different habitat types of Slovakia
Source: Parasit Vectors. 2016 May 20;9:292. doi: 10.1186/s13071-016-1560-z (PMC4874003; doi:10.1186/s13071-016-1560-z)
Supplement: Additional file 1: Table S1. — Prevalence of Babesia spp. in questing Haemaphysalis concinna per site in 2011–2013. Table S2. Occurence of Babesia spp. in questing Ixodes ricinus in Bratislava and Fúgelka. Table S3. Occurence of Babesia spp. in questing Ixodes ricinus in 2011–2013. Table S4. Occurence of Babesia spp. in questing Ixodes ricinus males, females and nymphs. Table S5. Variables remaining in the best selected model for Babesia microti prevalence in rodents. Table S6. Occurence of Babesia, Candidatus N. mikurensis and Anaplasma phagocytophilum in questing Ixodes ricinus. Table S7. Accession numbers of Apicomplexa 18S rRNA gene sequences (PDF 238 kb) [file 13071_2016_1560_MOESM1_ESM.pdf]

Table S1 Prevalence of *Babesia* spp. in questing *Haemaphysalis concinna* per site in 2011–2013

		2011		2012		2013		Fisher's	Total	
Site		% (pos/ex)	95% CI	% (pos/ex)	95% CI	% (pos/ex)	95% CI	exact test	% (pos/ex)	95% CI
Bratislava	Nymphs	33.3 (3/9)	11.1–66.7	0.0 (0/3)	–	4.3 (1/23)	0.0–13.0	0.073	11.4 (4/35)	2.9–22.9
	Females	0.0 (0/4)	–	0.0 (0/1)	–	25.0 (1/4)	0.0–75.0	1.000	11.1 (1/9)	0.0–33.3
	Males	0.0 (0/7)	–	–	–	0.0 (0/5)	–	–	0.0 (0/12)	–
	Adults total	0.0 (0/11)	–	0.0 (0/1)	–	11.1 (1/9)	0.0–33.3	0.476	4.8 (1/21)	0.0–14.3
	Total	15.0 (3/20)	0.0–34.9	0.0 (0/4)	–	6.3 (2/32)	0.0–15.6	0.565	8.9 (5/56)	1.8–17.9
Fúgelka	Nymphs	0.0 (0/16)	–	0.0 (0/4)	–	0.0 (0/4)	–	–	0.0 (0/24)	–
	Females	0.0 (0/3)	–	0.0 (0/1)	–	0.0 (0/2)	–	–	0.0 (0/6)	–
	Males	33.3 (1/3)	0.0–100.0	0.0 (0/1)	–	0.0 (0/1)	–	1.000	20.0 (1/5)	0.0–60.0
	Adults total	16.7 (1/6)	0.0–50.0	0.0 (0/2)	–	0.0 (0/3)	–	1.000	9.1 (1/11)	0.0–27.3
	Total	4.5 (1/22)	0.0–13.6	0.0 (0/6)	–	0.0 (0/7)	–	1.000	2.9 (1/35)	0.0–8.6
Total		9.5 (4/42)	2.4–19.0	0.0 (0/10)	–	5.1 (2/39)	0.0–12.8	0.715	6.6 (6/91)	2.2–12.1

(pos/ex), number of positive/number of examined; 95% CI, confidence interval

Table S2 Occurence of *Babesia* spp. in questing *Ixodes ricinus* in Bratislava and Fúgelka

	Bratislava<br>% (pos Ba/pos total)	Fúgelka<br>% (pos Fu/pos total)
Babesia microti	27.3 (12/44)	72.7 (32/44)
Babesia venatorum	53.8 (14/26)	46.2 (12/26)
other *Babesia* spp.	87.5 (7/8)	12.5 (1/8)

pos, positive; Ba, Bratislava; Fu, Fúgelka

Table S3 Occurence of *Babesia* spp. in questing *Ixodes ricinus* in 2011–2013

	2011<br>% (pos 2011/pos total)	2012<br>% (pos 2012/pos total)	2013<br>% (pos 2013/pos total)
Babesia microti	50.0 (22/44)	38.6 (17/44)	11.4 (5/44)
Babesia venatorum	57.7 (15/26)	7.7 (2/26)	34.6 (9/26)
other *Babesia* spp.	62.5 (5/8)	0.0 (0/8)	37.5 (3/8)

pos, positive

Table S4 Occurence of *Babesia* spp. in questing *Ixodes ricinus* males, females, and nymphs

	Males<br>% (pos M/pos total)	Females<br>% (pos F/pos total)	Nymphs<br>% (pos N/pos total)
Babesia microti	4.5 (2/44)	9.1 (4/44)	86.4 (38/44)
Babesia venatorum	30.8 (8/26)	7.7 (2/26)	61.5 (16/26)
other *Babesia* spp.	37.5 (3/8)	0.0 (0/8)	62.5 (5/8)

pos, positive; M, males; F, females; N, nymphs

Table S5 Variables remaining in the best selected model for *Babesia microti* prevalence in rodents

	B	S.E.	Wald	df	P	Exp(B)
Genus			37.250	2	< 0.001	
Genus (1)	–4.142	0.742	31.163	1	< 0.001	0.016
Genus (2)	–5.755	1.194	23.217	1	< 0.001	0.003
Gender (1)	2.345	0.813	8.312	1	0.004	10.431
Constant	–1.438	0.656	4.801	1	0.028	0.237

Categorical variables codings: Genus (1), Mice, Genus (2), *Myodes*; Gender (1), Males; variable removed by backward method was site; B, parameter estimate; S.E., standard error; Wald, Wald statistic = test of significance of the regression coefficient; P, significance level; Exp(B), odds ratio for parameter B

Table S6 Occurence of *Babesia, Candidatus* N. mikurensis and *Anaplasma phagocytophilum* in questing *Ixodes ricinus*

	Bratislava<br>% (pos Ba/pos total)	Fúgelka<br>% (pos Fu/pos total)
Candidatus N. mikurensis	34.5 (20/58)	65.5 (38/58)
Anaplasma phagocytophilum	74.7 (145/194)	25.3 (49/194)
Babesia spp.	37.7 (23/61)	62.3 (38/61)

pos, positive; Ba, Bratislava; Fu, Fúgelka

Table S7 Accession numbers of Apicomplexa 18S rRNA gene sequences

Species	Source	Name of the isolate (number of analysed samples with identical sequences)	BP	Accession numbers
Babesia microti	questing *I. ricinus* nymph from Bratislava	N233B (11)	474	KU362887
	questing *I. ricinus* nymph from Fúgelka	N178F (27)	474	KU550676
	questing *I. ricinus* female from Fúgelka	F43F (4)	474	KU362888
	questing *I. ricinus* male from Bratislava	M51B (1)	474	KU550677
	questing *I. ricinus* male from Fúgelka	M8F (1)	474	KU550678
	rodent-attached *I. ricinus* larva from Bratislava	L275HB (5)	474	KU362889
	rodent-attached *I. ricinus* larva from Fúgelka	L27HF (6)	474	KU550679
	rodent-attached *I. ricinus* nymph from Bratislava	N263HB (8)	474	KU362890
	rodent-attached *I. ricinus* nymph from Fúgelka	N1HF (1)	474	KU550680
	rodent-attached *I. ricinus* female from Fúgelka	F2HF (1)	474	KU362891
	rodent-attached *H. concinna* larva from Fúgelka	L126HF (2)	474	KU362892
	rodent-attached *H. concinna* female from Bratislava	F262HB (1)	474	KU362893
	rodent-attached *H. concinna* female from Fúgelka	F1HF (1)	474	KU550681
	Apodemus flavicollis from Bratislava	AF122B (4 spleens, 3 blood, 3 lungs, 2 skin biopsies from ear)	474	KU362894
	Apodemus flavicollis from Fúgelka	AF63F (3 spleens, 3 blood, 2 lungs, 1 skin biopsy from ear)	474	KU550682
	Microtus arvalis from Fúgelka	MA20F (6 spleens, 6 blood, 5 lungs, 6 skin biopsies from ear)	474	KU362895
	Myodes glareolus from Fúgelka	MG15F from blood (1)	474	KU362896
Babesia venatorum	questing *I. ricinus* nymph from Bratislava	N176B (7)	447	KU362897
	questing *I. ricinus* nymph from Fúgelka	N497F (9)	447	KU550683
	questing *I. ricinus* female from Bratislava	F56B (1)	447	KU550684
	questing *I. ricinus* female from Fúgelka	F1F (1)	447	KU362898
	questing *I. ricinus* male from Bratislava	M54B (6)	447	KU550685
	questing *I. ricinus* male from Fúgelka	M89F (2)	447	KU550686
	rodent-attached *I. ricinus* larva from Bratislava	L8HB (1)	447	KU362899
	rodent-attached *I. ricinus* nymph from Bratislava	N253HB (1)	447	KU362900
Babesia sp.	questing *I. ricinus* nymph from Fúgelka	N41F (1)	447	KU362901
	questing *I. ricinus* male from Bratislava	M340B (1)	447	KU362902
	rodent-attached *I. ricinus* larva from Bratislava	L302HB (1)	447	KU362903
Babesia canis	questing *I. ricinus* nymph from Bratislava	N30B (3)	447	KU362904
	questing *I. ricinus* male from Bratislava	M398B (2)	447	KU362905
Babesia odocoilei	questing *I. ricinus* nymph from Bratislava	N149B (1)	446	KU550687

Babesia sp. 2 (Eurasia)	questing *H. concinna* male from Fúgelka	M58F (1)	436	KU550688
	rodent-attached *H. concinna* larva from Bratislava	L189HB (1)	436	KU550689
Babesia sp. 1 (Eurasia)	questing *H. concinna* nymph from Bratislava	N141B (4)	436	KU550690
	questing *H. concinna* female from Bratislava	F117B (1)	436	KU550691
	rodent-attached *H. concinna* larva from Bratislava	L433HB (1)	436	KU550692
	rodent-attached *H. concinna* larva from Fúgelka	L244HF (1)	436	KU550693
	rodent-attached *H. concinna* larva from Bratislava	L355HB (2)	436	KU550694
Theileria sp.	questing *H. concinna* nymph from Bratislava	N419B (2)	467	KU550695
	rodent-attached *H. concinna* female from Fúgelka	F4HF (1)	467	KU550696
Sarcocystis sp.	*Microtus arvalis* from Fúgelka	MA128F skin biopsy from ear (4)	509	KU550697
	Myodes glareolus from Fúgelka	MG156 skin biopsy from ear (1)	501	KU550698
	Myodes glareolus from Fúgelka	MG223 skin biopsy from ear (1)	495	KU550699
